# Modeling for Prediction of Mortality Based on past Medical History in Hospitalized COVID-19 Patients: A Secondary Analysis

**DOI:** 10.1155/2024/3256108

**Published:** 2024-07-02

**Authors:** Seyyed Amir Yasin Ahmadi, Yeganeh Karimi, Arash Abdollahi, Ali Kabir

**Affiliations:** ^1^ Preventive Medicine and Public Health Research Center Psychosocial Health Research Institute Iran University of Medical Sciences, Tehran, Iran; ^2^ Tehran Heart Center Cardiovascular Diseases Research Institute Tehran University of Medical Sciences, Tehran, Iran; ^3^ Minimally Invasive Surgery Research Center Iran University of Medical Sciences, Tehran, Iran

## Abstract

**Introduction:**

Although COVID-19 is not currently a public health emergency, it will affect susceptible individuals in the post-COVID-19 era. Hence, the present study aimed to develop a model for Iranian patients to identify at-risk groups based on past medical history (PMHx) and some other factors affecting the death of patients hospitalized with COVID-19.

**Methods:**

A secondary study was conducted with the existing data of hospitalized COVID-19 adult patients in the hospitals covered by Iran University of Medical Sciences. PMHx was extracted from the registered ICD-10 codes. Stepwise logistic regression was used to predict mortality by PMHx and background covariates such as intensive care unit (ICU) admission. Crude population attributable fraction (PAF) as well as crude and adjusted odds ratio (OR) with 95% confidence interval (CI) were reported.

**Results:**

A total of 8879 patients were selected with 19.68% mortality. Infectious and parasitic diseases' history showed the greatest association (OR = 5.72, 95% CI: 4.20, 7.82), while the greatest PAF was for cardiovascular system diseases (20.46%). According to logistic regression modeling, the largest effect, other than ICU admission and age, was for history of infectious and parasitic diseases (OR = 3.089, 95% CI: 2.13, 4.47). A good performance was achieved (area under curve = 0.875).

**Conclusion:**

Considering the prevalence of underlying diseases, many mortality cases of COVID-19 are attributable to the history of cardiovascular disease. Future studies are needed for policy making regarding reduction of COVID-19 mortality in susceptible groups in the post-COVID-19 era.

## 1. Introduction

COVID-19 has been the most significant challenge to our healthcare systems in the modern era. With a total confirmed deaths of about 7 million in 771 million confirmed cases as of October 2023, it remains a challenge, with the number of cases still on the rise. The presentations of COVID-19 are heterogeneous with asymptomatic cases to patients transitioning from mild or moderate respiratory symptoms to the development of severe pneumonia, respiratory distress, multiple organ dysfunction, need for mechanical ventilation (MV), intubation, and ultimately death [1]. The equilibrium between the overall capacity of medical resources such as ICU beds and patients that are most at risk of developing severe diseases and death upon admission has placed pressure on the existing healthcare resources and underscored the need for a perfect triage [2].

The most frequently documented predictors of severe prognosis in COVID-19 patients encompass age, gender, findings derived from computed tomography (CT) scans, C-reactive protein levels, lactic dehydrogenase levels, and lymphocyte count [3]. Furthermore, patients with underlying diseases experience more unfavorable outcomes than those without such conditions. COVID-19 patients with a history of hypertension, obesity, chronic lung disease, diabetes, and cardiovascular diseases may lead to deteriorating outcomes more often than others [4].

Utilization of models that take into account several characteristics for estimating the possibility of having a poor prognosis could aid clinicians in prioritizing patients when allocating limited healthcare resources. Prediction models can incorporate predictors to estimate the probability of specific outcomes, aiding in risk stratification and personalized patient management [3]. However, the existing prediction models require refinement and validation to ensure their accuracy and applicability in the clinical setting. Nevertheless, due to methodological issues such as predictor bias, variability in defining COVID-19 cases (participants' bias), and evaluation of different outcomes (outcome measurement bias), a substantial proportion of the reported models are susceptible to a high risk of bias [3, 5]. Although COVID-19 is not currently a public health emergency, it will affect susceptible individuals in the post-COVID-19 era. Therefore, there is a need to have a statistical tool for identification of susceptible individual.

This study aims to develop a model for Iranian patients to identify at-risk groups based on the past medical history (PMHx) and demographic and some other clinical variables affecting the death of patients with COVID-19 hospitalized in the hospitals covered by the Iran University of Medical Sciences.

## 2. Methods

### 2.1. Study Design

A secondary study was conducted on the existed data using the existing data to design an exploratory model for prediction of death in COVID-19 patients. The study protocol and report were based on transparent reporting of a multivariable prediction model for individual prognosis or diagnosis (TRIPOD) statement. This study consisted of epidemiological study and statistical modeling. This study was confirmed by the Ethics Committee of Iran University of Medical Sciences with ethics registration number: IR.IUMS.REC.1400.388 at 26 July 2021.

### 2.2. Source of Data

A multicenter retrospective cohort registry known as the Medical Care Monitoring Center (MCMC) using the data recorded from the patients from the onset of the COVID-19 epidemic until April 2021 has been used in developing the model. All the patients hospitalized due to COVID-19 with ICD-10 code U07.1 and U07.2 in the mentioned time period were subjected for this study. Access to the data was through legal supervision of the Center of Statistics and Information Technology, Iran University of Medical Sciences. The management of the center shared the researchers an Excel file including all the variables of this study. In that dataset, the PMHxs were in one string variable including ICD-10 codes. Hence, the data cleaning process included creation of dummy variables for each PMHx, removing the observations with outlier ages, coding the texts for qualitative variables and managing the missing data.

### 2.3. Participants

This study includes patients admitted to affiliated hospitals of Iran University of Medical Sciences. Patients with COVID-19 are included in the study based on positive polymerase chain reaction (PCR) and/or involvement in pulmonary computed tomography (CT) scan. Patients under 18 years, people with negative test, and people with incompletely recorded information were excluded from this study.

### 2.4. Outcome

In this study, in-hospital mortality in patients with COVID-19 was considered as the main outcome. Therefore, hospitalized patients with COVID-19 who died due to the disease and had a positive CT scan or PCR test were included in the mortality group. Also, patients who recovered or were discharged from the hospital were included in the opposite group.

### 2.5. Predictors

The variables of age, gender, ICU admission, marital status (being single compared with married, divorced, or widowed), length of stay (LOS), and PCR result were included in the analysis. The underlying diseases of the patients were extracted and categorized using ICD-10 codes, and binary variables were created using the categories. These codes indicated any history of having these diseases resulting in hospital reference and hospitalization and were registered for the national ID of the patient at any time. The categories included are as follows.

  Infectious and parasitic diseases: A and B
  Neoplasms (malignancies): C.
  Diseases of the blood and blood-forming organs and specific disorders related to the immune system: D.
  Endocrine, nutritional, and metabolic diseases: E.
  Mental and behavioral disorders: F.
  Diseases of the nervous system: G.
  Eye diseases and ear and mastoid diseases: H.
  Diseases of the circulatory (cardiovascular) system: I.
  Diseases of the respiratory system: J.
  Diseases of the digestive system: K.
  Skin and subcutaneous tissue diseases: L.
  Diseases of the musculoskeletal system and connective tissue: M.
  Diseases of the genitourinary system: N.
  Pregnancy, childbirth, and puerperium: O.
  Some conditions caused by the perinatal period: P.
  Congenital malformations, deformities, and chromosomal abnormalities: Q.
  Injury, poisoning, and some other consequences of external causes: S and T.
  External causes of morbidity and mortality: V, X, and Y.

For blinding in the evaluation of the predictors, the categories were done using software commands and the researchers did not interfere in the categorization. The nomenclature mentioned above was based on the exact ICD-10 codes. In the present study, we integrated some codes and created new variable names as follows. The other variable names were the same.

  Infectious and parasitic diseases: AB.
  Hematologic disorders and neoplasms: CD.

### 2.6. Sample Size

For epidemiological study, sample size was calculated for Pearson chi-square test of association according to 0.2 probability of positive exposure (allocation ratio), 0.2 probability of positive outcome with 0.05 difference in positive exposure group, 0.05 two-tailed alpha error, and 0.95 power was obtained 6477. For statistical modeling, powerlog package of Stata software was used for estimation of sample size for logistic regression. Considering 0.2 and 0.25 outcome probabilities (such as assumptions in the epidemiological study), 0.05 two-tailed alpha error, 0.9 power, and squared multiple correlation 0.79 (equivalent to variance inflation factor (VIF) < 5), the sample size was obtained 4297 which could detect the significance of odds ratio (OR) = 1.333.

### 2.7. Missing Data

Complete-case analysis strategy was adopted as it was assumed that the missing data were completely at-random.

### 2.8. Statistical Analysis

For epidemiological study, descriptive statistics and crude tests (such as chi-square) were used. In addition, OR with 95% confidence interval (CI) and population attributable fraction (PAF) were reported based on direct calculation from two-by-two tables using *-cc-* command of the software. For statistical modeling, multiple logistic regression was used with backward Wald stepwise approach for model selection with maximum *P* value 0.1. Postestimation receiver operating characteristics (ROC) analysis was considered to study model performance, and the area under curve (AUC) was reported with 95% CI. In addition, for each predicted probability cutoff point of the model, sensitivity, specificity, correct classification, likelihood ratio (positive and negative), positive predictive value (PPV), negative predictive value (NPV), and Youden's J were reported. A nomogram was designed for practical use of the model. To evaluate the predictive ability of the estimated model, cross-validation was used with *-cvauroc-* package of the software. All the statistical processes of this study were conducted in Stata 17 (Stata Corp. LLC, TX, US).

### 2.9. Sensitivity Analysis

In order to consider the complexity modeling, we conducted model selection based on Akaike information criterion (AIC) and Bayesian information criterion (BIC) using *-aic_model_selection-*package of the software. The best models based on AIC and BIC were reported. Finally, the performances of all the models were compared as a sensitivity analysis along with a cross-validation for each model.

## 3. Results

### 3.1. Patients

A total of 8879 patients were selected from MCMC according to the inclusion and exclusion criteria and removal of missing data. At first, there were 9416 cases that 511 cases were removed due to personal consent for discharge or hospital transfer and escape from hospital. 25 cases were removed due to age less than 18 years. There was only one observation with missing datum which had no age. Therefore, it was considered as at-random missing datum and removed from analyses.

### 3.2. Epidemiological Study

**Figure 1:**
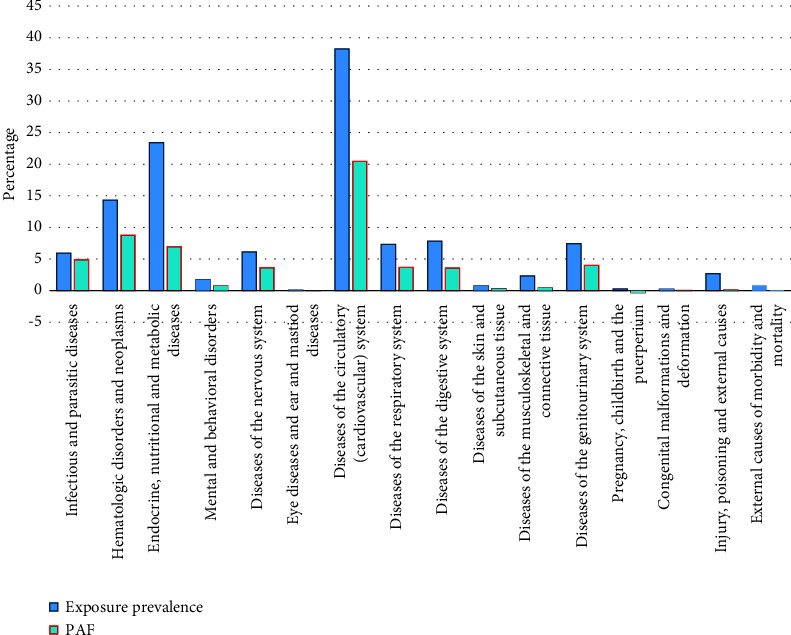
Comparison of exposure prevalence (equivalent to sensitivity in diagnostic tables) and PAF for each PMHx among the mortality cases. The amounts are in percentage, and the negative PAFs indicate preventive fraction. PAF: population attributable fraction; PMHx: past medical history.

There was 19.68% mortality among these patients. According to the cross-sectional design of this study, this outcome prevalence might be representative of the population in the studied place and time period. Therefore, all the statistical results were on the basis of this 19.68% prior probability. About 54.98% of the cases were 60-year-old and more. Demographic characteristic of the participants and the crude associations are shown. In brief, the infectious and parasitic diseases showed the greatest association (OR = 5.72, 95% CI: 4.20, 7.82), while the greatest PAF was for the cardiovascular system diseases meaning that 20.46% of COVID-19 deaths were attributable to the cardiovascular system diseases (Table 1). The chart of PAFs is shown in Figure 1. Accordingly, there should be two ideal conditions: (1) the PAF should be similar to the proportion of dead cases who had the mentioned diseases (called sensitivity in the chart). It meant that all the deaths were attributable to the mentioned diseases. In addition, these amounts should be large enough than other similarities. (2) Having a considerable PAF in spite of having a difference with sensitivity. Regarding the first ideal condition, infectious and parasitic diseases seemed to be the best variable as also it showed the greatest association. Regarding the second condition, diseases of the cardiovascular system showed a considerable amount of PAF.

### 3.3. Statistical Modeling

Logistic regression modeling was used for prediction of death in hospitalized COVID-19 patients. According to the enter method for model selection, no pairwise-collinearity (largest *r* = 0.468) and no multicollinearity (largest VIF = 1.30) was observed. As the model strategy was predicted, stepwise model selection with backward Wald method was used. According to the stepwise process of model selection, 12 covariates could significantly predict the outcome (*P* < 0.1). The largest effect, other than ICU admission and age, was for the history of infectious and parasitic diseases (OR = 3.089, 95% CI: 2.13, 4.47) (Table 2).

**Figure 2:**
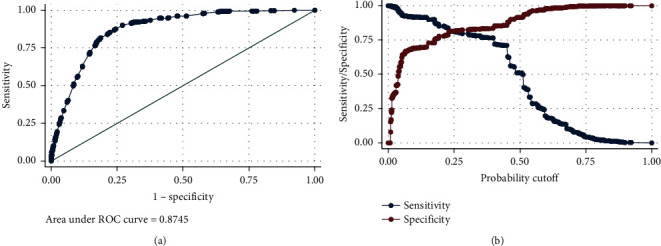
Model performance for the study multiple logistic regression (Table 2): (a) ROC curve and (b) sensitivity/specificity plot.

This model showed a suitable goodness-of-fit considering all the observed covariate patterns (*P*=0.152, chi-square = 388.61, and degrees of freedom = 361). However, Hosmer and Lemeshow test showed lack of a goodness-of-fit for most of the quantiles (*P*=0.012 for ten quantiles, the largest *P*=0.251 for three quantiles). In addition, the model performance was good (AUC = 0.875, 95% CI: 0.866, 0.883) (Figure 2). The result of cross-validation showed a similar performance (AUC = 0.873, standard deviation = 0.011). Considering predicted probability more than 0.5 as the predicted positive outcome, the model sensitivity was 50.83%, the model specificity was 91.38%, and the correctly classification rate was 83.40%. Considering the observed base rate of this study (19.68%), the positive and negative predictive values were 59.08% and 88.35%, respectively. The results of other cutoff points for predicted probability are shown (Table 3). The highest correct classification rate was for predicted probability greater than 0.5, while the largest Youden's J was for a predicted probability greater than 0.3. Therefore, considering the observed prevalence of mortality (19.68%), the best cutoff point was 0.5, while without considering this prevalence, the best cutoff point was 0.3 (as correct classification is influenced by prior probability, while Youden's J is not influenced). Although the specificities were higher than the sensitivities, the NPVs were achieved higher than the PPVs due to low prior probability. Therefore, this model was better for ruling out the high-risk cases.

**Figure 3:**
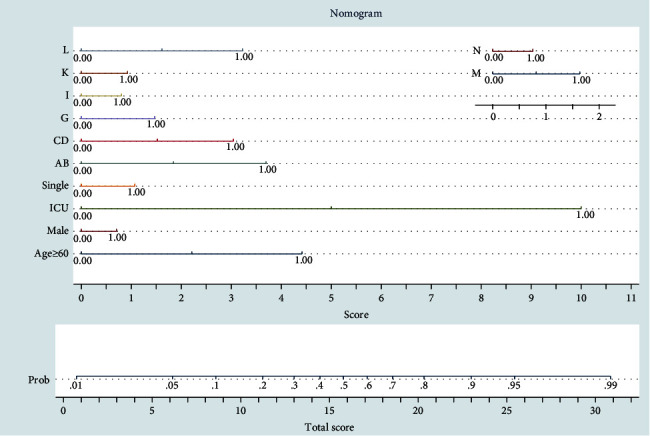
Nomogram for prediction of death probability based on logistic regression (Table 2). AB: infectious and parasitic diseases (combination of A or B ICD-10 codes), CD: hematologic disorders and neoplasms (combination of C or D ICD-10 codes), G: diseases of the nervous system, I: diseases of circulatory (cardiovascular) system, K: gastrointestinal diseases, L: skin and cutaneous tissue diseases, M: diseases of musculoskeletal system and connective tissue, and N: diseases of genitourinary system. All the covariates are binary with acceptable amounts of zero (negative) and one (positive).

This model can be used by both regression equation and nomogram (Figure 3). In the nomogram, each covariate has a scoring system. The total score of all covariates for each covariate pattern can be converted to probability. For a practical instance, a female patient (zero score) older than 60 years (about 4.5 scores) who is ICU admitted (about 10 scores) without any underlying disease (zero score) has about 0.4 probability of death considering total score 14.5.

### 3.4. Sensitivity Analysis

The final model was considered as the entry for model selection based on AIC and BIC. For each scenario of model selection, AUC and cross-validation results were reported. Hence, the scenario of AIC model selection showed a similar model with the full entry model (AUC = 0.875, cross-validation range: 0.862–0.890). The scenario of BIC model selection showed a model with four covariates less (AUC = 0.873, cross-validation range: 0.859–0.888) (supplementary table S1).

## 4. Discussion

The main objective of this study was to develop a model for Iranian patients to identify at-risk groups based on the PMHxs affecting the death of COVID-19 hospitalized patients. In this regard, we developed a predictive model for COVID-19 mortality using the real data obtained from MCMC. This model utilizes logistic regression to assess the risk of hospital mortality among COVID-19 patients based on their demographic information and comorbidities. We prioritized risk factors using the PAF index. Overall, the practical goal of this study was to present a user-friendly model to the clinicians to help the at-risk patients.

Our results indicated that ICU admission was significantly associated with an increased probability of mortality. However, it is important to note that ICU admission is more of a proxy for severity of illness and should be used primarily for prediction purposes rather than causal inference. From our analysis of demographic data and comorbidities, we found that history of infectious and parasitic diseases had the highest association with mortality, followed by malignancies and hematologic disorders, neurologic disorders, genitourinary diseases, cardiovascular diseases, and respiratory disorders, considering crude (unadjusted) analysis. It is essential to clarify that these associations represent the strength of the relationship, not the prevalence of the risk factors. Therefore, we used the PAF index to make the results more practical for the population.

In our multivariable regression, after adjusting for various factors, ICU admission and patient age ≥ 60 remained strong predictors of mortality. In addition, infectious and parasitic diseases, hematologic and malignancies, and skin disorders emerged as the most significant comorbidities associated with increased mortality risk. The difference between crude and multivariable analyses can be attributed to the role of each risk factor in predicting mortality. Given the study's purpose, covariates are not necessarily considered confounding variables, and the study is more suitable for prediction rather than establishing causality. From the comorbidities, cardiovascular disorders, malignancies, endocrine disease, and infectious and parasitic diseases had the highest PAF values, respectively. However, the prevalence of exposure to cardiovascular disorders, endocrine disease, and malignancies were higher, respectively. The drop in PAF against prevalence of exposure to the risk factor was due to the high prevalence of the disease, such as endocrine disease, besides a weak association with mortality (high prevalence and low OR), and vice versa. Although some diseases such as infectious and parasitic were strongly associated with mortality, they were not prevalent in the hospitalized patients (low prevalence and high OR). The differences between PAF and exposure prevalence are shown for each PMHx in Figure 1. Also, high PAF besides high exposure prevalence represents a significant association with mortality. For instance, cardiovascular disorders and malignancies, being prevalent in the study population, play critical roles in COVID-19 mortality. On the other hand, less prevalent diseases such as infectious and parasitic diseases exhibit low PAF values despite their strong association with mortality. Health policymakers can utilize this approach of using the PAF index to prioritize risk factors in the population.

In concluding this section, to make practical use of this predictive model for mortality, variables with both high OR and high PAF should be selected. Such factors are significantly associated with mortality, are prevalent in the population, and have a high PAF. For example, cardiovascular disorders meet these criteria. Variables with high OR are better suited for personal decision-making, while PAF is more useful for predicting mortality at the population level. This difference is due to the necessity of the prevalence of exposed cases in the population, in contrast to the necessity of a strong association between individual comorbidities and mortality. With an AUC of 0.875 and a probability threshold of >0.5 as a positive outcome, this model demonstrates good performance. It can be applied to assess an individual's risk of mortality based on their comorbidities.

Previously, some other studies reported PAF of different underlying diseases for COVID-19 mortality. In general, 35.7% of COVID-19-related deaths at all ages are attributable to chronic diseases [6]. Nguyen et al. studied the PAF of different underlying medical conditions in 87,526 hospitalized cases of COVID-19 in the US. Similar to our study, they found the highest PAF for cardiovascular diseases as it was responsible for 45% of COVID-19 deaths [7]. However, the present study was performed in a more homogenous population (all patients were Iranian, while the study of Nguyen et al. included different races) and found 20.46% of PAF.

Several recent studies have also developed prediction models for COVID-19 mortality. For example, Hajifathalian et al. in a study on 664 patients in the US, used logistic regression to show that age, ethnicity, hypertension, cardiovascular disorders, chronic kidney disease, and several other factors were significantly associated with 14-day mortality. Their model achieved an AUC of 0.86 for seven-day mortality and 0.83 for 14-day mortality [8]. The present study showed a similar performance (AUC = 0.875) with a larger sample size; however, the models' content was not similar as they had an exploratory approach. In other words, such modeling approaches on this topic would result in similar AUC in the approximate range of 0,8−0.9. Berenguer et al. developed a prediction model with an AUC of 0.82 for 30-day mortality using age, low age-adjusted SaO_2_, neutrophil-to-lymphocyte ratio, eGFR by the CKD-EPI equation, dyspnea, and sex. In addition, comorbidities like hypertension, obesity, liver cirrhosis, chronic neurological disorders, active neoplasia, and dementia were associated with increased risk of COVID-19 mortality; however, they were not included in this model. Moreover, the strongest association with increased mortality risk was observed in higher age categories (OR = 56.3 for age >90 years versus OR = 1 for age < 40 years). Notably, this study population had a higher median age 70 years in contrast to our study (median age = 61) [9]. In spite of differences in approaches and model covariates, such studies had an approximately similar model performance considering logistic regression modeling.

However, there were some studies with better model performance and accuracy in terms of modeling methods other than that logistic regression. According to the study of Gao et al. (2020), a highly accurate (AUC ranging from 0.9186 to 0.9762) ensemble model was developed using logistic regression, support vector machine (SVM), gradient boosted decision tree (GBDT), and neural network (NN) which was validated both internally and externally. Eight features including consciousness, male sex, sputum, blood urea nitrogen (BUN), respiratory rate (RR), D-dimer, number of comorbidities, and age were found as strong risk factors for mortality. In the mentioned study, considering threshold 0.6, AUC was calculated from 0.92 to 0.97 for the logistic regression model with an accuracy of 87.1% to 95.4% [10]. The role of comorbidities was defined better in a Cox proportional hazards' model and logistic regression model built by Moon et al. for predicting 30-day and 60-day mortalities (AUC = 0.959). Diabetes mellitus, cancer, and dementia as underlying diseases were significantly associated with 30-day and 60-day COVID-19 mortalities which supports our findings. Moreover, age ≥ 70, male sex, and presence of fever and dyspnea at the time of the COVID-19 diagnosis were reported as significant risk factors in this study [11]. Importantly, a statistically inspired modification of a partial least square (SIMPLS)-based model with high accuracy (AUC of 0.91 to 0.95, *Q*2 = 0.24) found coronary artery disease (CAD) has the highest predictive value for in-hospital mortality. Similar to other studies, diabetes, age > 65, altered mental status, dementia, and SaO_2_ < 88% are the other important risk factors [12]. Some other machine learning models including deep neural networks (DNN), random forest classifier (RF), eXtreme gradient boosting classifier (XGB), and SVM were utilized in a prediction model by Wan et al. In the mentioned study, age, income/personal property, long-standing illness, disability, and heart disorders were significantly associated with death in COVID-19 patients [13].

This study had some limitations. First of all, it was a secondary study on patients' registry, and data gathering was beyond our control. However, no human interference in data collection, large sample size, and multicenter data were the advantages of this data collection method. In addition, selected variables were well-defined features reducing data collection bias. There were some concerns about overcontrol bias related to adding ICU admission to the model that impeded a causal inference. It should be noted that there is no causality relation between ICU admission and death, and therefore, it may not be an intermediate of a causation chain. Based on the prediction approach of this model selection strategy, there is no concern for using this variable. Another limitation of logistic regression models is showing predictors regardless of low PAF. So, it would not be useful enough for public health. Hence, we reported the PAFs as well. The last limitation was not using train and test validation. Instead, we preferred to use all the available data as the main sample for analysis along with cross-validation. The strength of this study was low risk of overfitting in comparison to more complex models. Using logistic regression instead of advanced machine learning techniques and a large sample size might reduce the risk of overfitting. However, P-hacking in the stepwise process was inevitable.

## 5. Conclusions

In conclusion, age ≥ 60, male sex, single marital status, comorbidities (including infectious and parasitic, hematologic and malignancies, neurologic, cardiovascular, gastrointestinal, skin and subcutaneous, musculoskeletal and connective tissue, and urogenital), and ICU admission are important predictors of mortality with an accuracy of more than 85%. Although there were a variety of advanced machine learning models, logistic regression is user friendly and easy to interpret as our audiences were clinicians. This study can be used in personal and public clinical decision-making to reduce COVID-19 mortality. Future studies are needed for policy making regarding reduction of COVID-19 mortality in susceptible groups in the post-COVID-19 era. This consists of developing guidelines and policy briefs for prevention and treatment of COVID-19 in specific groups.

## Figures and Tables

**Table 1:** Characteristics of the COVID-19 patients and association with mortality.

Variable (unit)	*N* (percentage)/mean (SD)/median (IQR)			Association	
	All (*n* = 8879)	Survived (*n* = 7132)	Dead (*n* = 1747)	*P* value (test)	OR (95 (%)CI)/PAF
Age (year)^*∗*^	61.00 (17.26)	58.27 (16.56)	72.19 (15.46)	<0.001 (T)	
Male sex	4782 (53.89)	3756 (52.66)	1026 (58.73)	<0.001 (chi)	1.28 (1.15, 1.42)/12.81
ICU admission	3753 (42.27)	2155 (30.22)	1598 (91.47)	<0.001 (chi)	24.77 (20.71, 29.64)/87.78
Single marital status	3450 (38.86)	2762 (38.73)	688 (39.38)	0.61 (chi)	1.03 (0.92, 1.15)/1.07
LOS (day)^*∗∗*^	7 [5–10]	6 [5–9]	9 (4–15)	<0.001 (U)	
PCR positive	2856 (32.19)	2065 (28.95)	793 (45.39)	<0.001 (chi)	2.04 (1.83, 2.27)/23.14
PMHx					
Infectious and parasitic diseases	182 (2.05)	78 (1.09)	104 (5.95)	<0.001 (chi)	5.72 (4.20, 7.82)/4.91
Hematologic disorders and neoplasms	684 (7.70)	434 (6.09)	250 (14.31)	<0.001 (chi)	2.58 (2.18, 3.05)/8.76
Endocrine, nutritional, and metabolic diseases	1670 (18.81)	1261 (17.68)	409 (23.41)	<0.001 (chi)	1.42 (1.25, 1.62)/6.96
Mental and behavioral disorders	98 (1.10)	68 (0.95)	30 (1.72)	0.006 (chi)	1.81 (1.14, 2.84)/0.77
Diseases of the nervous system	292 (3.29)	185 (2.59)	107 (6.12)	<0.001 (chi)	2.45 (1.90, 3.14)/3.62
Eye diseases and ear and mastoid diseases	6 (0.07)	5 (0.07)	1 (0.06)	0.85 (chi)	0.82 (0.02, 7.30)/0.01
Diseases of circulatory (cardiovascular) system	2262 (25.48)	1594 (22.35)	668 (38.24)	<0.001 (chi)	2.15 (1.92, 2.41)/20.46
Diseases of the respiratory system	398 (4.48)	270 (3.79)	128 (7.33)	<0.001 (chi)	2.01 (1.60, 2.51)/3.68
Diseases of the digestive system	451 (5.08)	314 (4.40)	137 (7.84)	<0.001 (chi)	1.85 (1.49, 2.28)/3.60
Diseases of the skin and subcutaneous tissue	43 (0.48)	30 (0.42)	13 (0.74)	0.08 (chi)	1.77 (0.85, 3.51)/0.32
Diseases of the musculoskeletal and connective tissue	177 (1.99)	136 (1.91)	41 (2.35)	0.24 (chi)	1.24 (0.85, 1.77)/0.44
Diseases of the genitourinary system	387 (4.36)	257 (3.60)	130 (7.44)	<0.001 (chi)	2.15 (1.72, 2.68)/3.98
Pregnancy, child birth, and the puerperium	50 (0.56)	45 (0.63)	5 (0.29)	0.08 (chi)	0.45 (0.14, 1.13)/0.35
Congenital malformations and deformation	19 (0.21)	15 (0.21)	4 (0.23)	0.88 (chi)	1.09 (0.26, 3.42)/0.02
Injury, poisoning, and external causes	169 (1.90)	122 (1.71)	47 (2.69)	0.007 (chi)	1.59 (1.10, 2.25)/0.10
External causes of morbidity and mortality	66 (0.74)	52 (0.73)	14 (0.80)	0.75 (chi)	1.10 (0.56, 2.02)/0.07

^*∗*^Reporting mean and SD according to approximately normal distribution; ^*∗∗*^ reporting median and IQR according to the right-skewness of the distribution; SD: standard deviation; IQR: interquartile range; T: independent *t* test for variables with normal distribution; U: Mann–Whitney *U* test for variables with skewed distribution; chi: Pearson chi-square for comparison of qualitative variables; OR: odds ratio; CI: confidence interval; PAF: population attributable fraction (this is a preventive fraction in cases with OR <1); ICU: intensive care unit; LOS: length of stay; PCR: polymerase chain reaction.

**Table 2:** Multiple logistic regression model for prediction of death in the hospitalized COVID-19 patients.

Predictor	Odds ratio	Standard error	*z*	*P* value	95% CI (lower and upper)	
Age ≥ 60	3.851	0.295	17.58	<0.001	3.313	4.475
Male sex	1.239	0.082	3.25	0.001	1.089	1.410
ICU admission	21.158	1.937	33.33	<0.001	17.682	25.317
Single marital status	1.388	0.094	4.83	<0.001	1.215	1.585
Infectious and parasitic diseases	3.089	0.584	5.97	<0.001	2.133	4.474
Hematologic disorders and neoplasms	2.535	0.275	8.56	<0.001	2.049	3.136
Diseases of the nervous system	1.566	0.241	2.92	0.004	1.159	2.117
Diseases of the circulatory (cardiovascular) system	1.279	0.092	3.42	0.001	1.111	1.473
Diseases of the digestive system	1.326	0.174	2.15	0.031	1.026	1.715
Diseases of the skin and subcutaneous tissue	2.675	1.260	2.09	0.037	1.063	6.732
Diseases of the musculoskeletal and connective tissue	1.648	0.375	2.2	0.028	1.056	2.574
Diseases of the genitourinary system	1.260	0.171	1.7	0.088	0.966	1.644
Constant	0.008	0.001	−41.2	<0.001	0.006	0.010

CI: confidence interval; ICU: intensive care unit.

**Table 3:** Diagnostic accuracy indices and their normal 95% CIs for prediction of death for each cut-point of the predicted probability.

Cutoff point probability	Sensitivity	Specificity	Correct classification	LR+	LR−	PPV	NPV	Youden's J
≥0.3	0.795 (0.776, 0.813)	0.824 (0.815, 0.833)	0.818 (0.810, 0.826)	4.512 (4.268, 4.769)	0.249 (0.227, 0.274)	0.525 (0.506, 0.544)	0.942 (0.937, 0.948)	0.618
≥0.4	0.720 (0.698, 0.741)	0.852 (0.844, 0.860)	0.826 (0.818, 0.834)	4.869 (4.572, 5.185)	0.329 (0.305, 0.355)	0.544 (0.524, 0.564)	0.925 (0.919, 0.932)	0.572
≥0.5	0.508 (0.485, 0.532)	0.914 (0.907, 0.920)	0.834 (0.826, 0.842)	5.895 (5.395, 6.440)	0.538 (0.513, 0.565)	0.591 (0.566, 0.616)	0.884 (0.876, 0.891)	0.422
≥0.6	0.190 (0.172, 0.208)	0.977 (0.974, 0.981)	0.822 (0.814, 0.830)	8.315 (6.945, 9.955)	0.829 (0.810, 0.848)	0.671 (0.629, 0.712)	0.831 (0.823, 0.839)	0.167
≥0.7	0.077 (0.065, 0.090)	0.994 (0.992, 0.996)	0.813 (0.805, 0.822)	12.247 (8.776,17.092)	0.929 (0.916, 0.941)	0.750 (0.687, 0.813)	0.815 (0.807, 0.823)	0.071

CI: confidence interval; LR: likelihood ratio; PPV: positive predictive value; NPV: negative predictive value.

## Data Availability

The data that support the findings of this study are available on request from the corresponding author. The data are not publicly available due to privacy or ethical restrictions.

## Supplementary Materials

Supplementary Materials Supplementary Table S1 shows sensitivity analysis for model selection and cross-validation.
