# Supplementary material for: Modeling for Prediction of Mortality Based on past Medical History in Hospitalized COVID-19 Patients: A Secondary Analysis
Source: Can J Infect Dis Med Microbiol. 2024 Jul 2;2024:3256108. doi: 10.1155/2024/3256108 (PMC11233185; doi:10.1155/2024/3256108)
Supplement: Supplementary Materials — Supplementary Table S1 shows sensitivity analysis for model selection and cross-validation. [file 3256108.f1.pdf]

Supplementary Table 1. Sensitivity analysis for model selection and cross-validation

Model 1. AIC-based model considering our final backward model (table 2) as the entry

Stata output:

```
. aic_model_selection logistic Death age_60 Gender ICU MaritalName AB CD G I
K L M N
      AIC Model
 8142.335 Death age_60
 8123.388 Death age_60 Gender
 6053.873 Death age_60 Gender ICU
 6039.115 Death age_60 Gender ICU MaritalName
 5996.750 Death age_60 Gender ICU MaritalName AB
 5936.281 Death age_60 Gender ICU MaritalName AB CD
 5929.862 Death age_60 Gender ICU MaritalName AB CD G
 5920.541 Death age_60 Gender ICU MaritalName AB CD G I
 5918.084 Death age_60 Gender ICU MaritalName AB CD G I K
 5915.458 Death age_60 Gender ICU MaritalName AB CD G I K L
 5912.774 Death age_60 Gender ICU MaritalName AB CD G I K L M
 5911.881 Death age_60 Gender ICU MaritalName AB CD G I K L M N
```

Impression: The best model (smallest AIC) is highlighted which is the same with our final model (table 2).

AUC: 0.875

Cross-validation:

```
. cvauroc Death age_60 Gender ICU MaritalName AB CD G I K L M N, graph
1-fold (N=888).........AUC =  0.862
2-fold (N=888).........AUC =  0.870
3-fold (N=888).........AUC =  0.873
4-fold (N=888).........AUC =  0.887
5-fold (N=888).........AUC =  0.867
6-fold (N=888).........AUC =  0.890
7-fold (N=888).........AUC =  0.888
8-fold (N=888).........AUC =  0.862
9-fold (N=888).........AUC =  0.869
10-fold (N=887).........AUC =  0.866
Model:logistic
Seed:7777
--------------------------------------------------------------------
Cross-validated (cv) mean AUC, SD and Bootstrap Bias Corrected 95%CI
--------------------------------------------------------------------
cvMean AUC:                        | 0.8733
Bootstrap bias corrected 95%CI:    | 0.8642, 0.8819
cvSD AUC:                          | 0.0108
----------------------------------------------------------------
```

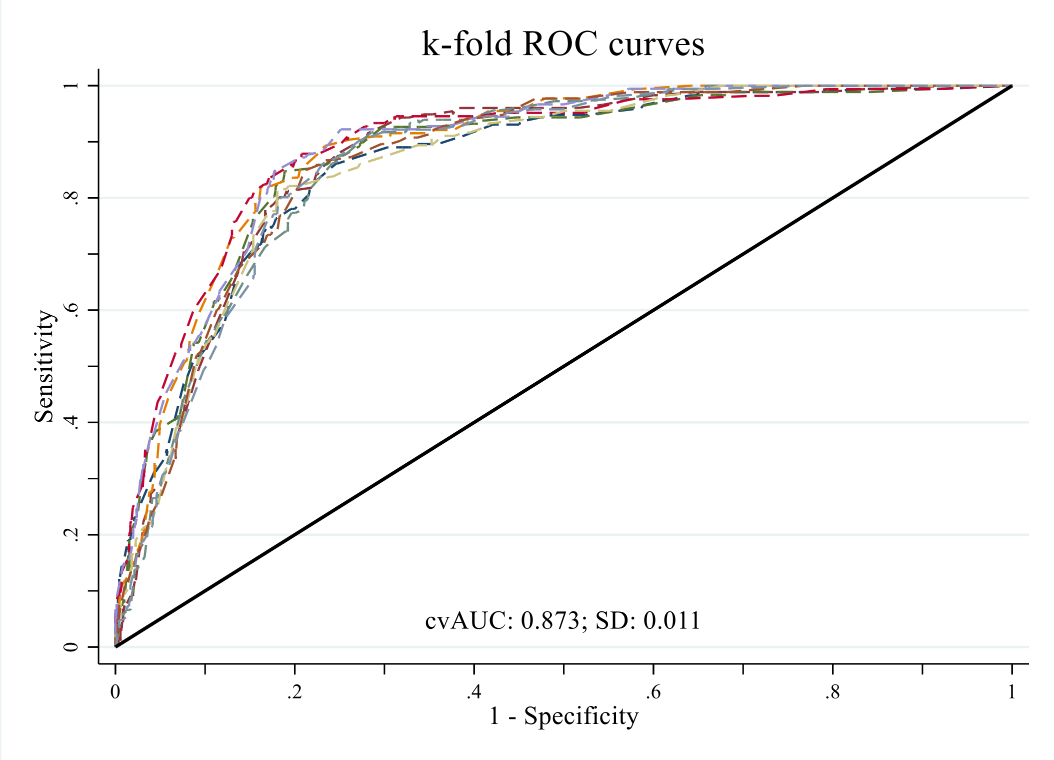

Model 2. BIC-based model considering our final backward model (table 2) as the entry

Stata output:

```
. aic_model_selection logistic Death age_60 Gender ICU MaritalName AB CD G I
K L M N, bic
      BIC Model
 8156.518 Death age_60
 8144.663 Death age_60 Gender
 6082.239 Death age_60 Gender ICU
 6074.572 Death age_60 Gender ICU MaritalName
 6039.299 Death age_60 Gender ICU MaritalName AB
 5985.921 Death age_60 Gender ICU MaritalName AB CD
 5986.593 Death age_60 Gender ICU MaritalName AB CD G
 5984.364 Death age_60 Gender ICU MaritalName AB CD G I
 5988.998 Death age_60 Gender ICU MaritalName AB CD G I K
 5993.464 Death age_60 Gender ICU MaritalName AB CD G I K L
 5997.871 Death age_60 Gender ICU MaritalName AB CD G I K L M
 6004.070 Death age_60 Gender ICU MaritalName AB CD G I K L M N
```

Impression: The best model (smallest AIC) is highlighted that has an inconsiderable difference with our final model (table 2) (5984 vs 6004).

AUC: 0.873

Cross-validation:

```
. cvauroc Death age_60 Gender ICU MaritalName AB CD G I, graph
1-fold (N=888).........AUC =  0.859
2-fold (N=888).........AUC =  0.867
3-fold (N=888).........AUC =  0.873
4-fold (N=888).........AUC =  0.887
5-fold (N=888).........AUC =  0.867
6-fold (N=888).........AUC =  0.888
7-fold (N=888).........AUC =  0.888
8-fold (N=888).........AUC =  0.865
```

```
9-fold (N=888).........AUC =  0.868
10-fold (N=887).........AUC =  0.865
Model:logistic
Seed:7777
-------------------------------------------------------------------
Cross-validated (cv) mean AUC, SD and Bootstrap Bias Corrected 95%CI
-------------------------------------------------------------------
cvMean AUC:                       | 0.8726
Bootstrap bias corrected 95%CI:   | 0.8630, 0.8807
cvSD AUC:                         | 0.0108
---------------------------------------------------------------
```

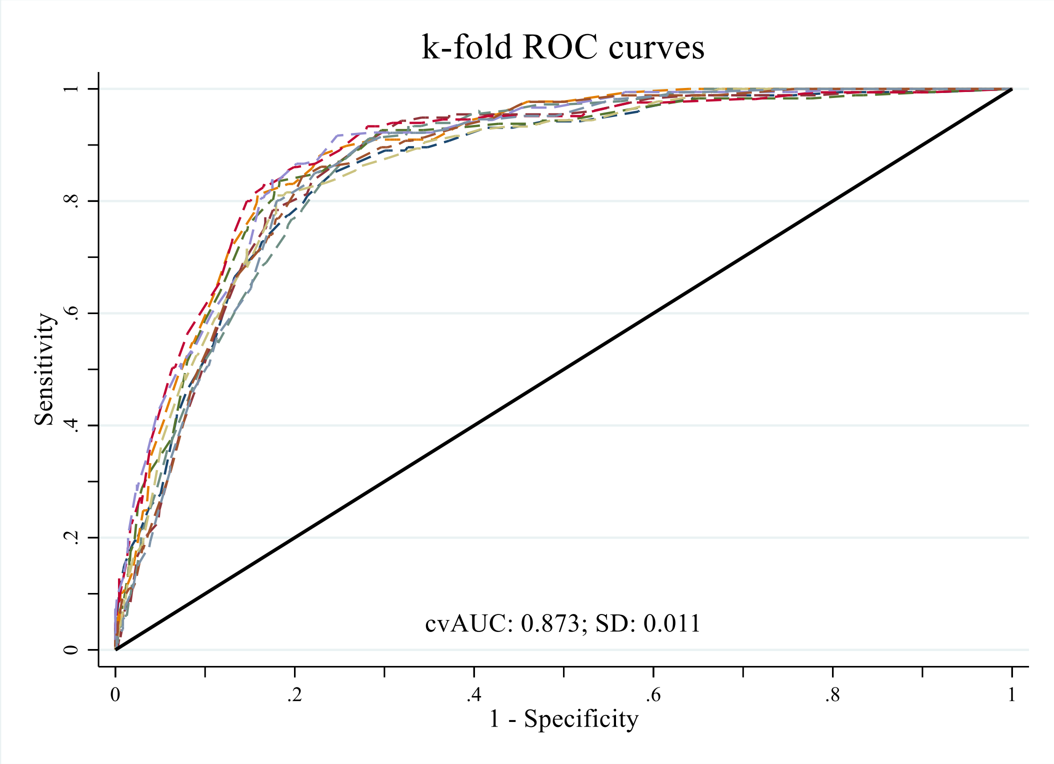

AB: infectious and parasitic diseases (combination of A or B ICD-10 codes), CD: hematologic disorders and neoplasms (combination of C or D ICD-10 codes), G: diseases of the nervous system, I: diseases of circulatory (cardiovascular) system, K: gastrointestinal diseases, L: skin and cutaneous tissue diseases, M: diseases of musculoskeletal system and connective tissue, N: diseases of genitourinary system.
